# Machine learning driven optimization of biomedical waste ash concrete for sustainable construction

**DOI:** 10.1371/journal.pone.0343424

**Published:** 2026-03-13

**Authors:** Christo George, Sathvik Sharath Chandra, Prashant M. Topalakatti, Vishnu R., Shahaji Patil, George Uwadiegwu Alaneme

**Affiliations:** 1 Department of Civil Engineering, Dayananda Sagar College of Engineering, Bengaluru, India; 2 KLE Institute of Technology, Hubballi, India; 3 Visvesvaraya Technological University, Belagavi, India; 4 Department of Civil Engineering, Kampala International University, Uganda; Graphic Era Deemed to be University, INDIA

## Abstract

Concrete produced using ash from biomedical waste is a sustainable construction solution that can help reduce the environmental impact associated with cement production. The study develops a database of Biomedical Waste Ash (BMWA) concrete from literature sources and applies advanced Machine Learning (ML) techniques to predict the mechanical properties of the mixes. Four ML models (Random Forest (RF), Self-Attention and Intersample Attention Transformer (SAINT), TabNet, and an Ensemble model) were subjected to performance evaluation, hyperparameter optimization, and ten-fold cross-validation. RF and TabNet achieved the highest predictive performance, with an R² of 0.82 across strength parameters, while SAINT demonstrated stable generalization but reduced accuracy for certain strength parameters. The ensemble model showed poorer performance than each model, which emphasizes the capability of robust standalone models in specific and limited databases. The external validation showed good agreement between them and hence supports the reliability of our models. Sustainability Index (SI) incorporates cement substitution, durability improvement, and retained strength to evaluate the overall performance of the BMWA concrete at 15% BMWA. The study proposes an integrated data-driven framework that combines advanced ML methods, interpretability analysis, external validation, and sustainability indexing to optimize BMWA concrete and indicate its dual role in reducing the environmental footprint of the cement industry and utilizing biomedical waste in building materials, thus supporting the principles of the circular economy and the global sustainability goals.

## 1. Introduction

Concrete is the most common building material globally, next to water, and is critical to the world’s-built environment because of its durability, versatility, and affordability. The massive quantities of concrete being produced worldwide, however, significantly contribute to climate change and pose a major strain on natural resources and ecosystems. Approximately 70% of total global CO₂ emissions resulting from cement production come from the manufacture of Ordinary Portland cement (OPC) itself, primarily from the calcination of limestone and the high-energy clinker formation process required to produce cement in high-temperature kilns [1–3]. The ongoing trend toward greater urbanization and increased infrastructure demand means that global cement usage will continue to grow, and therefore global greenhouse gas emissions from cement manufacturing will also continue to rise [4,5]. There is hence increasing interest in developing more sustainable types of binding agents and alternative materials to help reduce the reliance on OPC [6,7].

To reduce the environmental impact of OPC-based concrete, SCM (Supplementary Cementitious Material) is added to it. SCMs have proved to be effective in reducing the carbon footprint associated with the production of concrete and have also enhanced the durability and longer-term performance. The three most widely used SCMs are rice husk ash, fly ash, silica fume, and ground granulated blast furnace slag (GGBS), and numerous studies have demonstrated that these SCMs will enhance several of the characteristics of concrete, including reduced permeability, increased chemical resistance, and increased long term strength, Although SCM’s have been proven to be beneficial in numerous ways, the availability of traditional SCM’s can vary greatly depending on the location and thus, may not be readily available to all interested parties. Researchers have started utilizing non-conventional and less common waste streams, which present an environmental problem if not disposed of properly.

Studies have shown that waste ashes generated from various industrial and agricultural activities can perform well in concrete mixes and can be engineered to achieve desired mechanical and durability properties. For example, sugarcane bagasse ash has been effectively integrated into LC3-based systems and has provided enhancements in terms of flexural performance and durability in industrial environments containing high concentrations of CO₂, illustrating its potential as a beneficial SCM. Fly ash and pond ash have also been studied as possible replacements for cement and fine aggregate in concretes and have resulted in the modification of time-dependent phenomena such as shrinkage and the enhancement of volumetric stability and long-term performance. Biomedical waste ash (BMWA) is a particularly problematic and underutilized waste stream [8,9]. BMWA is generated daily from the incineration of gloves, syringes, masks, and other medical equipment. While incineration results in a reduction in the volume of waste of 80–90%, the resulting BMWA is frequently landfilled without adequate processing [10–12]. BMWA is a problem due to the presence of heavy metals and the very fine particle size of the ash, creating the possibility of leachate, contaminating groundwater, and air pollution [13,14].

The inclusion of BMWA in cementitious materials provides an alternative solution to disposal in landfills while reducing the consumption of cement [15,16]. When incorporated at controlled replacement levels, BMWA particles are expected to be physically entrapped within the cementitious matrix, which may reduce the mobility of potentially hazardous constituents [17–19]. However, the extent of immobilization depends on material composition, exposure conditions, and long-term durability performance. The majority of studies evaluating the incorporation of BMWA into cement paste have indicated that BMWA typically does not exhibit strong pozzolanic properties and acts primarily as an inert filler at moderate replacement ratios [20–23]. Therefore, the mechanical performance of the concrete will be mainly determined by physical properties, as opposed to chemical effects [24–26]. However, the higher the ratio of BMWA to cement, the greater the amount of cement dilution that occurs, and the lower the compressive strength of the concrete will be [27–29]. While moderate additions of BMWA to the mix may result in some loss of compressive strength, they have been shown to significantly improve durability (i.e., provide better resistance to chloride ion penetration) through improvements in particle packing and pore refinement [30–32]. The observations suggest potential suitability for durability-focused applications; however, practical implementation requires additional validation, particularly with respect to environmental safety and long-term performance. The present study focuses on predictive performance assessment rather than regulatory qualification or environmental compliance validation [33,34].

While the incorporation of waste ash into concrete is a relatively new concept, the feasibility of utilizing industrial and agricultural by-product materials in cementitious systems has been demonstrated in numerous studies [35,36]. Rice husk ash is effective in providing resistance to chloride penetration in marine environments. Sugarcane Bagasse Ash and Palm Oil Fuel Ash have also been demonstrated to exhibit some degree of pozzolanic activity at low replacement levels [37–39]. These findings emphasize the need for more sophisticated analytical tools capable of identifying the complex and nonlinear interactions that exist between the various components of waste-based concrete systems [40,41].

Studies on the use of Municipal solid waste incineration (MSWI) ash as an additive to secondary binders in concrete indicate that MSWI ash can be acceptable if controlled by manufacturing and processing methods, but the variability in the chemical composition of MSWI ash and the potential for heavy metal leachate to contaminate soil and water restricts MSWI ash’s acceptance as a replacement for the primary binder in traditional cement materials; likewise, variability is an issue with BMWA. There is also a lack of large-scale research on incorporating BMWA into concrete, most of which lacks predictive modelling techniques to evaluate the experimental results, resulting in empirical and regression-based models created to investigate BMWA concrete systems failing to account for non-linear behaviors occurring during the hydration process; as such, there is a need to develop new data-driven methods to model BMWA that can identify nonlinear interactions and complex relationships among features [42,43].

Due to their ability to identify the complexities in BMWA concrete systems, Machine Learning (ML) models have the potential to be used as tools to model these systems [44]. Regression models are limited by their assumption of a defined linear or non-linear relationship between the independent variables before training the model; in contrast, ML models do not make this assumption and instead learn the relationship between the independent and dependent variables based on the learned data [45–48]. There have been many investigations conducted on utilizing various types of ML models to predict various properties of BMWA concrete systems, including, but not limited to, compressive strength, flexural performance, workability characteristics, and durability indicators (such as carbonation depth and sulfate resistance) [49]. Results from previous studies have shown that the ANN, SVR, RF, and GB models have higher predictive accuracy compared to empirical models [50–53].

More recently, transformer-based and interpretable deep learning models, such as SAINT and TabNet, have been utilized for tabular data sets, exhibiting both excellent predictive capability and explainability through attention mechanisms to identify dominant input features [54–56]. The ensemble learning approaches have been employed to enhance the robustness and generality of the predictions [57–59]. Recent studies have developed hybrid data-driven concrete mix designs that integrate probabilistic learning and evolutionary optimization techniques [60,61]. Such hybrid frameworks combine Bayesian model updating with NSGA to allow for uncertainty-aware multi-objective optimization of steel fiber-reinforced concrete [62–65]. Analogous hybrid frameworks coupling ML and evolutionary algorithms have been applied to green concretes incorporating recycled aggregates, demonstrating the efficacy of predictive-optimization integration for sustainable concrete design [66–68].

The current study combines advanced ML models, interpretability techniques, and sustainability assessment to support a safe, optimized, and data-driven utilization of biomedical waste ash in concrete [10,69,70]. The integration of predictive modelling with interpretability analysis and an SI framework is consistent with the principles of circular economies and supports the development of sustainable and resilient infrastructure.

The primary objectives of the study are:

- To compile a literature-derived, harmonized dataset of BMWA concrete and apply advanced ML algorithms to predict their 28-day mechanical strength.
- To evaluate model performance through multi-metric assessment and k-fold cross-validation for accuracy and robustness.
- To use SHAP, sensitivity, and feature importance analyses to interpret predictions and identify data-driven optimal BMWA replacement levels, balancing strength and durability.

### 1.1. Research gap and significance

Despite being used extensively in conventional concrete technologies, the application of ML in relation to unconventional binders like BMWA is limited. The majority of the current research studies heavily rely on laboratory evaluation, which limits their potential to be scaled up and limits the generalizability of the findings based on a variety of mixture design configurations and environmental conditions. There is no comparative evaluation of attention-based and ensemble ML models for BMWA concrete within a unified interpretability and sustainability framework. This lack of computational integration only means that engineers and practitioners currently lack robust predictive frameworks to optimize BMWA mix designs and forecast durability.

The study addressed the existing gaps in research by incorporating literature source data with the use of advanced ML predictive modeling to determine the mechanical properties of BMWA concrete. The use of this approach provides both the rigour of method that exists within current practices as well as the advantage of providing data-based models of safe and cost-effective mix design optimization. The study illustrates that BMWA has the potential to be used as an alternative to cement as a sustainable solution to waste management, green building, and the reduction of greenhouse gas emissions. The study supports the principles of the circular economy and promotes global sustainability objectives, including SDGs 11 (Sustainable Cities and Communities), 12 (Responsible Consumption and Production), and 13 (Climate Action).

## 2. Methodology

**Fig 1:**
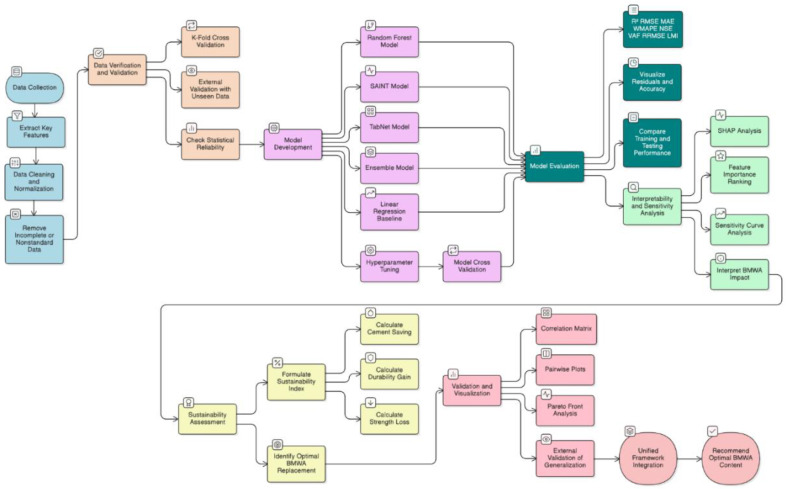
Methodology of the study.

The methodology in Fig 1 outlines the integration of data preprocessing, ML models, and sustainability assessment into a six-phase process (each phase represented in different colour codes).

- *Phase 1: Data Collection and Preprocessing:* Data collection is carried out through literature sources of 600 screened concrete mix design data points. Each data point includes features selected from available literature, and each input is normalized to create a consistent dataset.
- *Phase 2: Data verification and statistical validation:* Model validation was performed using k-fold cross-validation and external literature-derived datasets not used during training, ensuring unbiased evaluation without any laboratory experimentation. The external dataset primarily represents interpolation within the BMWA substitution domain rather than extrapolation
- *Phase 3: Development of Predictive Models:* Predictive models are developed using RF, SAINT, TabNet, and Ensemble Approaches with Hyperparameter Tuning and 10-Fold Cross-Validation. These four models were selected based on recent evidence demonstrating superior performance over traditional baseline algorithms (i.e., SVR, ANN, GB) for nonlinear concrete strength prediction.
- *Phase 4: Evaluation of Performance:* Performance evaluation is done using statistical measures (i.e., R², Root Mean Square Error (RMSE), Mean Absolute Error (MAE), Weighted Mean Absolute Percentage Error (WMAPE), Nash-Sutcliffe Efficiency (NSE), Variance Accounted For (VAF), Relative Root Mean Square Error (RRMSE), Loss Margin Index (LMI)), comparative analysis, and residual error plots.
- *Phase 5: Interpretation and Sensitivity Analysis:* Feature importance rankings, SHAP analysis, and sensitivity curves are used to assess interpretability and sensitivity of the models.
- *Phase 6: Sustainability Analysis:* A Sustainability Index is used to evaluate sustainability and balance cement savings, durability increases, and strength losses to determine the optimal BMWA mix ratio (10–15%) for use in sustainable building applications.

The methodology is systematic and provides accuracy, interpretability, and practical sustainability outcomes for the proposed method. The models are considered as advanced predictors relative to common baseline methods and provide a comprehensive basis to compare predictive capabilities in BMWA-based concrete. All data used in the study were obtained from published peer-reviewed literature sources. No new experimental or laboratory data were generated. Accordingly, reproducibility in the study refers to analytical and predictive reproducibility of data-driven trends rather than laboratory replication of individual concrete mix designs.

### 2.1. Machine learning

The mechanical properties of concrete (compressive strength, tensile strength, and flexural strength) at 28 days with exposure to the impacts of BMWA were used to create ML models. The primary goal was to establish the influence of BMWA as a substitute for cement on the strength parameters of the concrete that will be used to develop predictive models of concrete building materials and ultimately allow for their optimization. RF, TabNet, SAINT, Ensemble Model, and other supervised ML models were employed in the current study for predicting and sensitivity analysis. To evaluate how well each model fits the data, using evaluation metrics, the dataset was divided into two subsets for training and testing. Descriptive statistical analysis and visual representations were used to visually represent the data distribution and show which of the input parameters are sensitive to variations in the output parameters.

### 2.2. Dataset preparation

The dataset was compiled from screened literature-derived data. The literature data was adopted to enhance dataset diversity and capture a wider range of mix design variability, which is essential for training robust and generalizable ML models. Studies reporting standardized testing procedures, complete mix proportions, and compatible strength measurements were included, while datasets with missing information, inconsistent units, or non-standard testing methods were excluded. The dataset used concrete mixtures using BMWA as a substitute for cement. The strength values at 7, 14, and 28 days were extracted from the source studies to assess the Compressive Strength (CS), Tensile Strength (TS), and Flexural Strength (FS). Normalization was employed during the process of pre-processing of data to take into consideration the differences in scale among the input parameters, i.e., BMWA content (both percentage and kilograms per cubic meter), cement content, and strength values for curing ages. A high correlation (r = 0.96) existed between BMWA (%) and BMWA (kg/m^3^); a strong negative correlation (r = −0.93) also existed between BMWA (%) and cement content. The strength development at the three curing ages was found to be very similar; the intercorrelation coefficients of the CS measured at 7, 14, and 28 days were found to be in the range of 0.78 to 0.85.

### 2.3. Construction of the model development

To capture the complex interaction (nonlinear) between the content of BMWA (cement replacement) and the mechanical properties of concrete, four different ML models were selected. Each of the models (RF, SAINT, TabNet, and Ensemble Model) has unique and complementary methodological advantages that will enable a comprehensive evaluation of each model’s ability to predict from structured literature data. A reference benchmark has been provided for evaluating the performance of advanced ML models. A Multiple Linear Regression (LR) model was implemented as a baseline. The LR model was trained using the same input features, normalization procedure, and 10-fold cross-validation protocol as the other models. This baseline enabled assessment of whether nonlinear and attention-based models offer meaningful performance gains over traditional linear modeling approaches commonly used in concrete strength prediction.

#### 2.3.1. Random forest.

Random Forest is an ensemble learning method based on decision trees. It constructs multiple trees using bootstrap samples of the dataset and introduces randomness by selecting a subset of features at each node split. The final prediction is obtained by averaging the outputs of all trees (for regression tasks). RF reduces the potential for overfitting and provides a consistent and reliable performance with the noisy data that is generated by an experiment. The ability to account for non-linear interactions among input variables makes RF ideal for materials science applications, particularly when input variables (e.g., cement, BMWA content, and curing time) are expected to interact in non-linear ways. In addition, the high degree of predictability achieved through the use of RF in this study (mean R² = 0.82 with denormalized RMSE values reported in the Results section) was due to the ability of RF to capture both linear and non-linear relationships within the data.

$\hat{y}=\frac{\text{1}}{T}\sum_{t=\text{1}}^{T}\;h_{t}(x)$ for Regression


$$\hat{y}=\text{arg}\;{max}_{c\in C}\sum_{t=\text{1}}^{T}\text{1}\left\{h_{t}(x)=\text{C}\right\}$$

where, T = number of trees, $h_{t}(x)$ = the class predicted by the t-^th^ tree for input x, C = the set of possible classes.

#### 2.3.2. Self-attention and intersample transformer.

The SAINT model is a transformer-based deep learning model developed for use with tabular data. In contrast to previous models that treated each row as independent, SAINT has two types of attention mechanisms: self-attention to learn about feature relationships inside one sample, and inter-sample attention to learn about sample relationships. These mechanisms enable SAINT to identify complex patterns of relationships between features that would not have been captured by decision trees alone. SAINT can automatically identify hierarchical feature interactions; this ability is particularly useful in materials and concrete-related datasets, where microstructural and material-level effects are combined. In addition to identifying global feature dependencies, SAINT avoided overfitting and was strong in generalizing from the training data to the testing data (R² = 0.784, VAF = 77.18%).


$$Q=XW^{Q},\;K=XW^{K},\;V=XW^{V}\;$$

$$SelfAttention(X)=softmax\;\left(\frac{QK^{T}\;}{\sqrt{d_{k}}}\right)V$$

$$Q_{batch}=X_{batch}W^{Q},\;K_{batch}=X_{batch}W^{K},\;V_{batch}=X_{batch}W^{V}\;$$

$$IntersampleAttention\left(X_{batch}\right)=softmax\;\left(\frac{Q_{batch}K_{batch}^{T}\;}{\sqrt{d_{k}}}\right)V_{batch}$$

where, $W^{Q},W^{K},W^{V}\in R^{d\times d_{k}}$ are learned projection matrices, $d_{k}$ is the dimension of the keys/queries.

#### 2.3.3. TabNet.

TabNet is a framework in the area of deep learning that has been designed particularly to work with structured table-based data sets. TabNet uses a sequential-attention approach to focus on the most important features at each step in the prediction process, which allows for an analogy between how humans evaluate information and how a model evaluates a set of input information. The masks used by TabNet are also interpretable, so that while the model is producing results, it also indicates which variables are most influential in those results. The primary reason that TabNet lends itself to concrete research is that it will show how individual features (such as the amount of BMWA within a material or the value of early-age compressive strength) influence the prediction process at various steps. In the present study, TabNet was shown to have performance characteristics comparable to Random Forest (R² = 0.82; RMSE = 0.185 MPa). The feature interpretation aspects of TabNet were found to be consistent with the purpose of the study to connect BMWA content to strength reductions.


$$\hat{y}=\sigma \left(\sum_{t=\text{1}}^{T}W_{t}f_{t}\right)$$

where, $W_{t}$ are learned weights, σ is an activation function (like softmax for classification).

#### 2.3.4. Ensemble model.

The ensemble model used averaging to combine the predictions of all of the base learners (TabNet, SAINT, and RF). The idea behind using an ensemble model was to use multiple learners to improve stability and reduce variance. In most cases, ensembles are superior to single models. In this specific dataset, however, the averaging effect reduced the accuracy of the strongest models (RF and TabNet) (training r-squared: 0.724, testing R^2^: 0.713). Although the ensemble had a lower accuracy than the standalone models, including the ensemble in the models was methodologically important; it provided evidence that the ensemble did not improve the performance of the application. Therefore, it demonstrated that for specialized materials datasets, stronger standalone models may outperform hybridized combinations.


$${\hat{y}}_{\text{ensemble }}=\frac{\text{1}}{k}\sum_{i=\text{1}}^{k}\;{\hat{y}}_{i}$$

where, ${\hat{y}}_{\text{ensemble }}$ is the final prediction from the ensemble, k is the number of models in the ensemble, ${\hat{\;y}}_{i}$ is the prediction from the i-th individual model.

### 2.4. Performance evaluation

The models predictive capability was examined via various statistical methods to obtain a general understanding of all of its predictive capabilities. R² measured the proportion of variation in strength data that the model’s predictions accounted for, rather than relying on one or two statistics. The WMAPE is expressed as a percentage and thus is easier to understand and compare with other applications of engineering. NS, VAF, RSR, and LMI are additional measures used to examine the stability and reliability of each model. The use of several measures provided a comprehensive examination of each model’s predictive capability, including its accuracy, precision, and reliability.

Coefficient of Determination (R²):


$$R^{\text{2}}=\text{1}-\frac{\sum \;{\left(y_{i}-{\hat{y}}_{i}\right)}^{\text{2}}}{\sum \;{\left(y_{i}-\bar{y}\right)}^{\text{2}}}$$

Root Mean Square Error (RMSE):


$$RMSE=\sqrt{\frac{\text{1}}{n}\sum \;{\left(y_{i}-{\hat{y}}_{i}\right)}^{\text{2}}}$$

Mean Absolute Error (MAE):


$$MAE=\frac{\text{1}}{n}\sum \;\left|y_{i}-{\hat{y}}_{i}\right|$$

Weighted Mean Absolute Percentage Error (WMAPE):


$$WMAPE=\frac{\sum \;\left|y_{i}-{\hat{y}}_{i}\right|}{\sum \;y_{i}}\times \text{100}$$

Nash-Sutcliffe Efficiency (NS):


$$NS=\text{1}-\frac{\sum \;{\left(y_{i}-{\hat{y}}_{i}\right)}^{\text{2}}}{\sum \;{\left(y_{i}-\bar{y}\right)}^{\text{2}}}$$

Variance Accounted For (VAF):


$$VAF=\left(\text{1}-\frac{\text{var}(y-\hat{y})}{\text{var}(y)}\right)\times \text{100}$$

Relative Standard Error Ratio (RSR):


$$RSR=\frac{RMSE}{STDEV_{obs}}$$

Linear Model Index (LMI):


$$LMI=\frac{\sum \;\left(y_{i}-\bar{y}\right)\left({\hat{y}}_{i}-\overset{―}{\hat{y}}\right)}{\sqrt{\sum \;{\left(y_{i}-\bar{y}\right)}^{\text{2}}\sum \;{\left({\hat{y}}_{i}-\overset{―}{\hat{y}}\right)}^{\text{2}}}}$$

### 2.5. Hyperparameter selection

The best hyperparameter of each of the models was found through systematic tuning. To trade-off bias and variance in RF, the number of estimators (trees), maximum depth, and minimum samples per split were varied. The final configuration (n_estimators = 200, max_depth = 10, min_samples_split = 2) produced consistent predictions. For SAINT, the number of attention heads, transformer layers, and embedding dimension was tuned to minimize RMSE without overfitting. TabNet required optimization of decision steps, relaxation factor, and learning rate; the tuned configuration ensured efficient sequential attention while maintaining interpretability. The ensemble model was constructed by averaging predictions from the tuned base learners. Grid search and random search methods were used to do hyperparameter tuning, where validation performance was used to guide the process.

This ensured that each algorithm was evaluated under its most competitive configuration for the given dataset. The process of hyperparameter tuning of all four models is indicated in Table 1, which indicates the search ranges explored, the best value found, and the strategies applied. Tuning of the RF and TabNet was primarily performed by grid search, whereas SAINT used a random search to explore its much larger parameter space. The ensemble was concluded manually and constructed by a simple average of RF, SAINT, and TabNet, which showed that the best stable predictions were obtained.

**Table 1:** Hyperparameter tuning details and final configurations.

Model	Hyperparameter	Search Range Explored	Optimized Value	Tuning Strategy
**RF**	Number of estimators (n_estimators)	50–500 (step 50)	200	Grid Search
	Maximum depth (max_depth)	5–20	10	Grid Search
	Minimum samples per split	2–10	2	Grid Search
**SAINT**	Attention heads	2–12	8	Random Search
	Transformer layers	2–10	6	Random Search
	Embedding dimension	32–128 (step 16)	64	Random Search
**TabNet**	Decision steps (n_steps)	3–10	5	Grid Search
	Relaxation factor (γ)	1.0–2.0	1.5	Grid Search
	Learning rate	0.001–0.05	0.02	Random Search
**Ensemble**	Base learners included	RF, SAINT, TabNet,	RF + SAINT + TabNet	Manual Selection
	Combination method	Weighted average/ Simple average	Simple average	Manual Selection

Note: All optimized hyperparameters reported in Table 1 were selected based on cross-validated performance within the training folds only.

### 2.6. Cross-validation (k-fold)

Standard k-Fold Cross-Validation (k = 10) was utilized to evaluate how cross-validation affects both the robustness and ability of models to generalize. Each of the 10 folds of the dataset was approximately 10% of the total dataset, with each fold being utilized as a test set once throughout the cross-validation process. Therefore, for each pass through the process, 9 of the 10 folds were utilized to train the model, while the tenth fold was utilized to test the trained model. The process was completed 10 times; thus, every sample was utilized once for testing and nine times for training. Standard Deviation represents variability between models; the mean ± standard deviation of the performance metrics is the average performance metric value of all ten iterations. Cross-validation also helps prevent over-fitting of the models and provides an accurate representation of what can be expected from the models when they are presented with a new data set, versus the accuracy of the models based upon a specific train-test split. Before model training, input features and output targets (compressive, tensile, and flexural strengths) were normalized to improve numerical stability and learning efficiency of the ML algorithms. Model predictions were subsequently inverse-transformed to their original physical units before performance evaluation. Unless stated otherwise, all error metrics reported in this study are expressed in physical units (MPa) after denormalization.

Hyperparameter optimization was carried out within the training folds of the 10-fold cross-validation framework to avoid information leakage. Grid search was employed for RF and TabNet models due to their relatively structured hyperparameter spaces, while random search was adopted for the SAINT model to efficiently explore its higher-dimensional parameter space. For each fold, optimal hyperparameters were selected exclusively based on training data performance, and the corresponding validation fold was used only for unbiased model evaluation. Bayesian optimization was not applied in the study. A summary of the cross-validation process and its implementation is shown in Table 2.

**Table 2:** k-Fold cross-validation setup and implementation.

Parameter	Description
Number of folds (k)	10
Training/Validation ratio	90% training, 10% validation per fold
Iterations per model	10 (each fold used once for validation)
Evaluation metrics	R², RMSE, MAE, WMAPE, NS, VAF, RSR, LMI
Reporting method	Mean ± standard deviation of metrics across all folds
Purpose	To reduce overfitting, assess model robustness, and ensure reproducibility

### 2.7. Heatmap analysis

**Fig 2:**
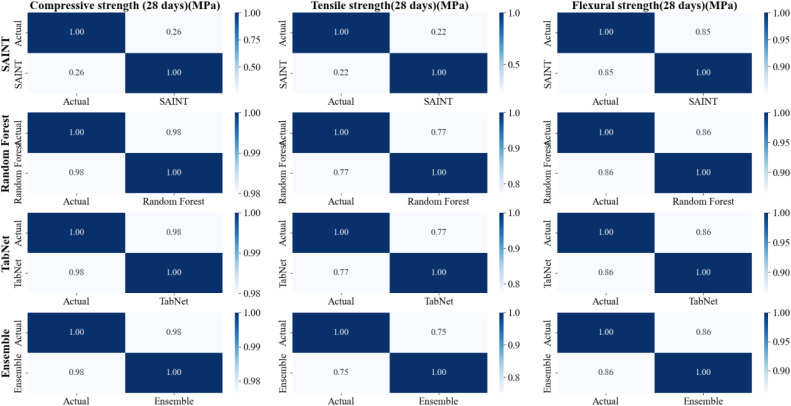
Correlation heatmaps between actual and predicted 28-day compressive, tensile, and flexural strengths for ML models.

The heatmap in Fig 2 depicts the agreement between real and projected values for three essential mechanical parameters of concrete after 28 days: compressive strength, tensile strength, and flexural strength. Each row represents one of the assessed models (SAINT, RF, TabNet, and Ensemble), with off diagonal values representing the correlation between actual and predicted strength. The RF and TabNet models correspond very well with actual findings in all three attributes, with values regularly over 0.98, demonstrating their resilience. SAINT, on the other hand, had a worse agreement for compressive and tensile strength, with off-diagonal values (0.22–0.26) indicating significant deviations from real values.

Flexural strength was better captured by all models, including SAINT, with off-diagonal values closer to 0.85–0.86, indicating greater reliability than compressive and tensile strength. The Ensemble model was steady but slightly less consistent than RF and TabNet, which could be attributed to the averaging effect diluting the strengths of various models. The heatmap shows that RF and TabNet make the most accurate and consistent predictions across all strength metrics, whereas SAINT struggled with compressive and tensile strength and does well for flexural strength. The investigation demonstrated that ensemble learning does not always outperform powerful standalone models on this dataset.

### 2.8. Comparative model performance analysis

**Fig 3:**
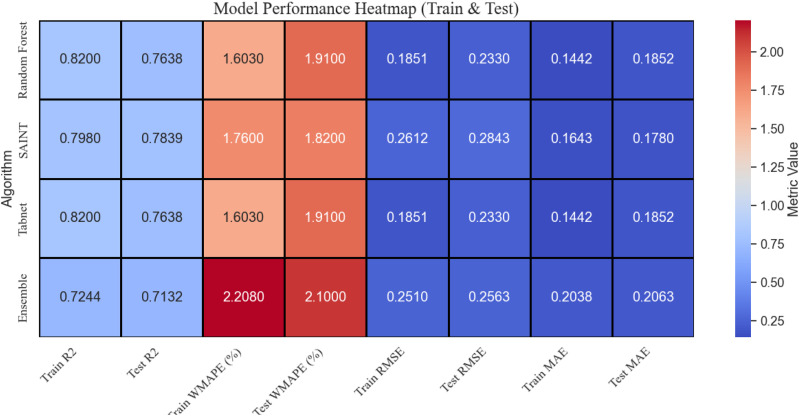
Model performance heat map for all models.

The detailed visual analysis of various ML models, such as RF, SAINT, TabNet, and Ensemble, in terms of such key evaluation indicators as R^2^, WMAPE (%), RMSE, MAE, and others, is shown in Fig 3. The performance of the model behaviour during the training and the generalization on the unseen data are easy to compare since the measures are presented individually in the training and testing data. To increase the visual distinction and clarity, all of the statistics are presented in the form of columns with a contrast colour gradient: bright pink in training metrics and brilliant blue in testing metrics. The numerical values are represented in every cell for easy interpretation.

The data structure is highlighted by the black gridlines that divide the cells, which make it simple to see trends or abnormalities quickly. The colour gradient provides an intuitive sense of relative performance: darker colours represent better performance (e.g., higher R² or lower error), while lighter colours indicate weaker performance. The heatmap can quickly observe how closely the models generalize, where overfitting might occur (high training but low test performance), and which models are most consistent. The visualization was particularly useful for decision-making when selecting the most reliable model for deployment based on balanced performance across all metrics. The baseline LR model demonstrated markedly lower predictive performance than all advanced ML models employed in this study. Across the evaluated mechanical properties, the LR model yielded R² values in the range of approximately 0.52–0.56, with corresponding RMSE values of about 2.1–2.4 MPa for compressive strength. This level of error was substantially higher than that of RF, SAINT, and TabNet, indicating that linear formulations were insufficient to capture the nonlinear interactions between biomedical waste ash replacement, cement content, and strength development. These results confirmed the necessity of using nonlinear and attention-based learning architectures for reliable prediction and optimization of BMWA concrete performance.

### 2.9. Correlation matrix

**Fig 4:**
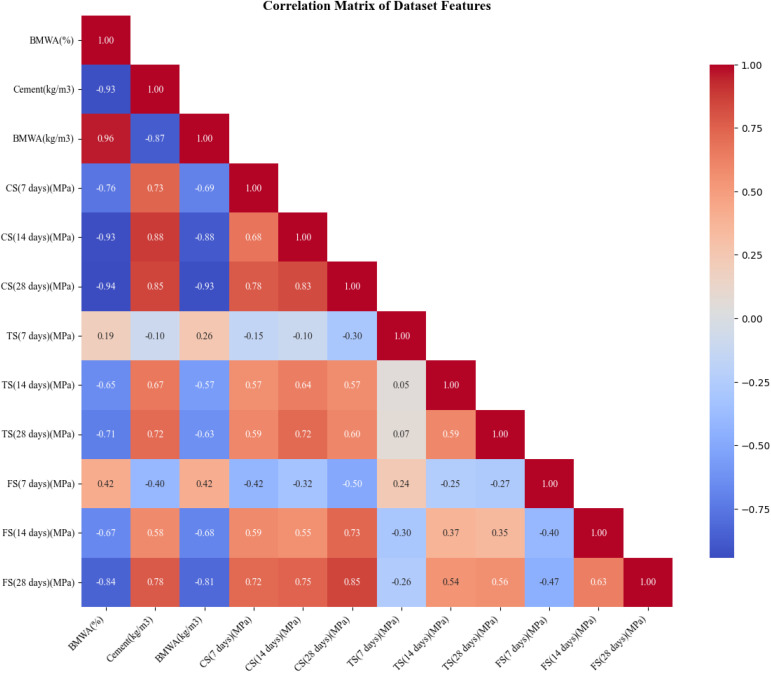
Correlation matrix.

A correlation matrix for the characteristics of BMWA is shown in Fig 4. A very strong positive relationship existed between BMWA% and kg/m³ BMWA%, where an almost perfect positive correlation of 0.96 was found, as they both relate to the same material. There was a strong negative correlation (0.93) between BMWA% and cement content; as BMWA% increases, cement content will decrease. As expected, due to the nature of testing for compressive strength over time, there was a strong positive correlation between compressive strength at all tested curing ages (7, 14, and 28 days) (0.78–0.85) to demonstrate a consistent pattern of developing compressive strength within all mixes.

The tensile and flexural strength had positive correlations with compressive strength; however, those correlations were generally less than 0.60, and therefore, while there was some association between these tests, it was not particularly strong. Siddiqui et al. (2025) stated that the three strength measurements could be used to determine the overall mechanical properties of concrete. However, tensile strength measured at 7 days had lower correlation coefficients with the other two strength measures, indicating that tensile strength at 7 days may exhibit greater variability and therefore be more sensitive to conditions existing at early curing times. The strength measurements had negative correlations with % BMWA, further supporting the idea that using too much BMWA in concrete can result in weakened concrete, consistent with previous research results. The matrix showed the trade-offs that exist when attempting to maximize both mechanical performance and replace cement with BMWA. The matrix also demonstrated that while tensile, flexural, and compressive strength may have some differences, they are still closely related and provide reliable methods for determining the quality of concrete.

### 2.10. Scatter plot

**Fig 5:**
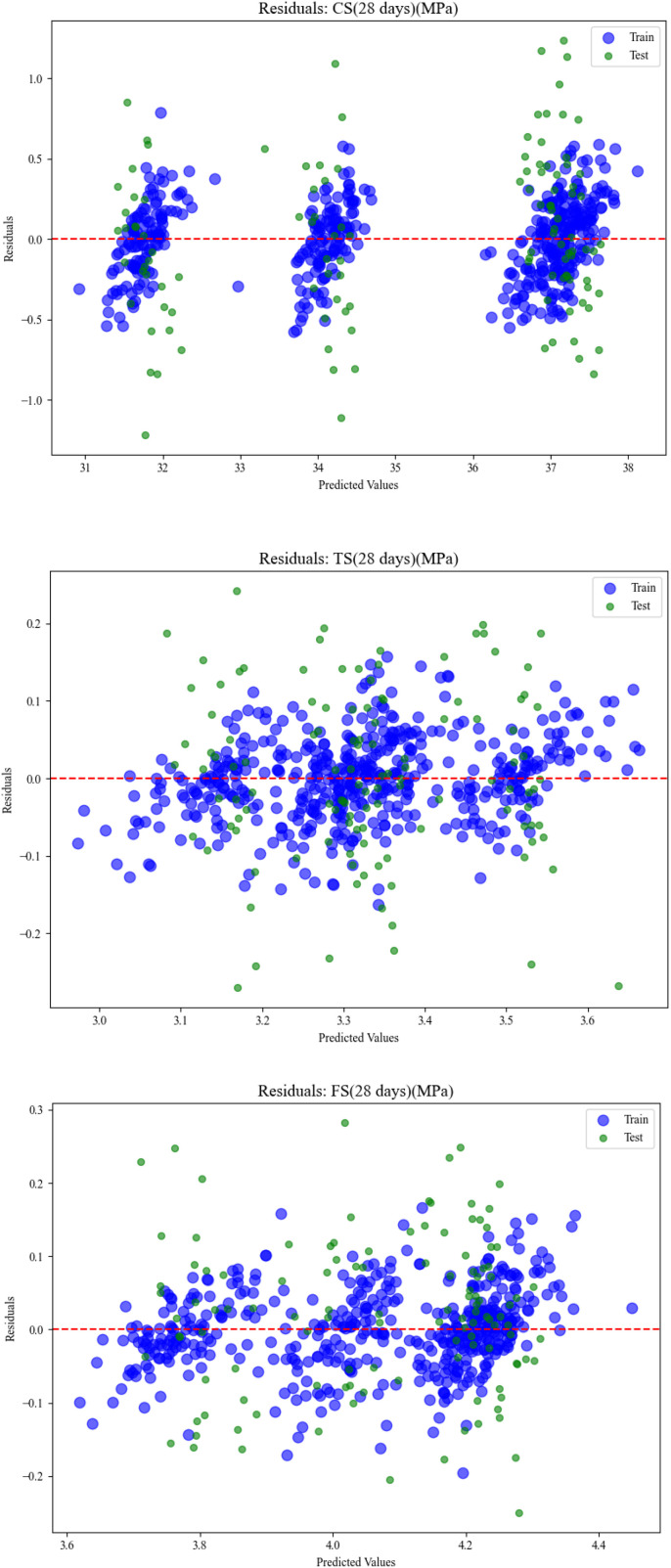
Scatter plots for predicted values of train and test of CS, TS, and FS.


The scatter in Fig 5 depicts the residuals of the ML model for each target variable, CS, TS, and FS after 28 days [47]. The residuals are the difference between what we see in the data (the actual value) and what the model would have been able to predict (the predicted value). This residual plot has the predicted values on the X-axis and Residuals (Actual Value – Predicted Value) on the Y-axis. Blue dots on this residual plot show the residuals of the training data, and green dots show the residuals of the testing data. There is a red dashed horizontal line at Y = 0 shown for reference; if the forecasts were exact matches with the true values, then they would lie directly on this line.

$$e_{i}=y_{i}-\hat{y_{i}}$$

The residual analysis is used to evaluate the model performance and identify potential flaws such as bias, heteroscedasticity, and outliers. Ideally, residuals should be randomly distributed around the zero line, indicating that the model is learning the underlying patterns with no systematic errors. A pattern or trend in residuals may indicate difficulties such as heteroscedasticity or model underfitting. The separation of colour and marker size improves clarity in distinguishing between train and test data behaviour, allowing for a simple visual comparison of how well the model generalizes to new data.

### 2.11. Sustainability index development

SI relates to both the mechanical performance and durability improvement, and environmental issues are evaluated collectively within the context of BMWA concrete. The SI is based on the three parameters; first parameter is the loss in compressive strength compared to the control mix; second parameter is the durability gain, which is quantified as the decrease in chloride permeability as determined by the Rapid Chloride Permeability Test (RCPT); third parameter is the cement savings that result from using BMWA instead of all cement in the mixture. As such, this formulation has the objective of minimizing strength loss while maximizing both cement savings and durability benefits to the service life of the structure. The SI was developed as an equally weighted composite index of environmental, durability, and structural performance (w₁ = w₂ = w₃ = 1/3) to provide an unbiased and balanced engineering assessment, thus eliminating any subjectivity that may occur when one or more of these sustainability criteria are given greater priority than others. Durability indicators used in the Sustainability Index were harmonized at the trend level rather than absolute magnitude, as they were compiled from heterogeneous literature sources; accordingly, durability performance is interpreted comparatively rather than as a standardized RCPT benchmark. The results in a single normalized indicator for comparing the sustainability of various levels of BMWA replacement and for determining those ranges of BMWA replacement that will produce significant environmental advantages while producing no unacceptable loss of structural performance.


SI = w1·(Cement Saving) + w2·(Durability Gain) − w3·(Strength Loss)

## 3. Results and discussion

### 3.1. Descriptive statistics

Descriptive statistics is a branch of statistics that involves the description and arrangement of data in a way that helps in its usefulness. It provides easy numerical summaries and graphs to describe the key characteristics of a dataset, such as mean and median, standard deviation and variance, minimum and maximum, skewness, and kurtosis. The descriptive statistics in Table 3 help to determine patterns, trends, and variability of the data, making it easier to understand and apply more complicated analyses. The descriptive statistics were employed to investigate the input properties (cement, fine aggregate, coarse aggregate, and biomedical waste ash) and output properties (compressive, tensile, and flexural strength) so as to gain insight about data distribution and correlation. It should be noted that fine aggregate and coarse aggregate contents were maintained constant across all mix designs; consequently, they exhibit zero variance and are included only for completeness of mix design description, while they do not contribute to variability-based statistical interpretation or model learning.

**Table 3:** Descriptive analysis.

Feature	Mean	Median	Std Dev	Variance	Min	Q1 (25%)	Q3 (75%)	Max	Skewness	Kurtosis	Effect Size (Cohen’s d)
Cement(kg/m^3^)	344.435	344.42	14.7064	216.2780	310.99	333.56	355.810	381.64	0.0097	−0.8335	0.0010
Biomedical waste ash(kg/m^3^)	27.466	26.89	13.4640	181.2781	2.00	14.997	39.7075	50.54	−0.0267	−1.5194	0.0428
Fine aggregate(kg/m^3^)	636.00)	636.00	0.0000	0.0000	636.00	636.00	636.000	636.00	NaN	NaN	NaN
Coarse aggregate(kg/m^3^)	1147.000	1147.00	0.0000	0.0000	1147.00	1147.0	1147.00	1147.00	NaN	NaN	NaN
Compressive strength (MPa)	35.040	35.53	2.3221	5.3921	30.56	32.97	37.1700	38.55	−0.3457	−1.3911	−0.2110
Tensile strength(MPa)	3.325	3.32	0.1646	0.0271	2.89	3.21	3.4400	3.77	0.0632	−0.3002	0.0335
Flexural strength(MPa)	4.066	4.11	0.2115	0.0447	3.51	3.90	4.2400	4.52	−0.4062	−0.7886	−0.2050

### 3.2. Feature importance analysis

**Fig 6:**
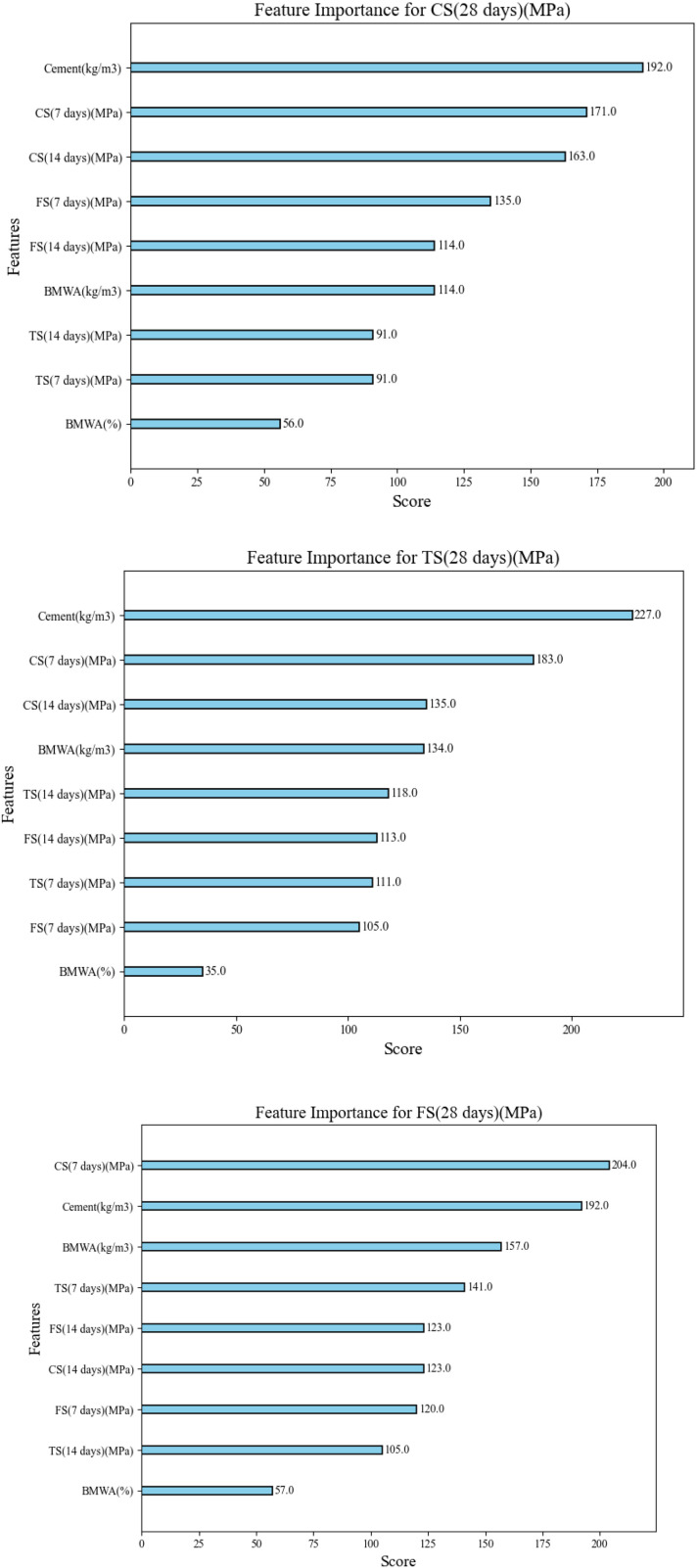
Plots of feature importance for CS, TS, and FS.

Fig 6 shows the correlation between the BMWA content and the mechanical properties of concrete. The greatest values of the best three values of mechanical strength were found using the control mixture (0% BMWA). There was an obvious trend toward a decrease in each measure of strength as the BMWA replacement ratio increased. The mechanical properties of the mixture are obviously degraded due to the substitution of cement by the addition of BMWA. Permeability decreases steadily as the percentage of BMWA in the mixture increases, with the lowest permeability (the highest resistance to chloride penetration) occurring when the mixture contained 15% BMWA. This indicates that although the addition of BMWA decreases the mechanical strength of the concrete, it increases its durability, an important finding for consideration in future uses of the mixture. The observed reduction in mechanical strength with increasing BMWA content is primarily attributed to cement dilution, as BMWA exhibits limited pozzolanic reactivity and functions predominantly as an inert filler. However, the concurrent improvement in durability-related indicators can be mechanistically explained by particle packing and pore refinement effects, wherein the fine BMWA particles partially fill capillary voids and reduce connectivity of pore networks. This microstructural densification restricts ion transport pathways, thereby improving resistance to chloride ingress despite reductions in load-bearing cementitious phases. The results highlight a fundamental strength–durability trade-off rather than a contradiction in material behaviour.

### 3.3. Box plot

**Fig 7:**
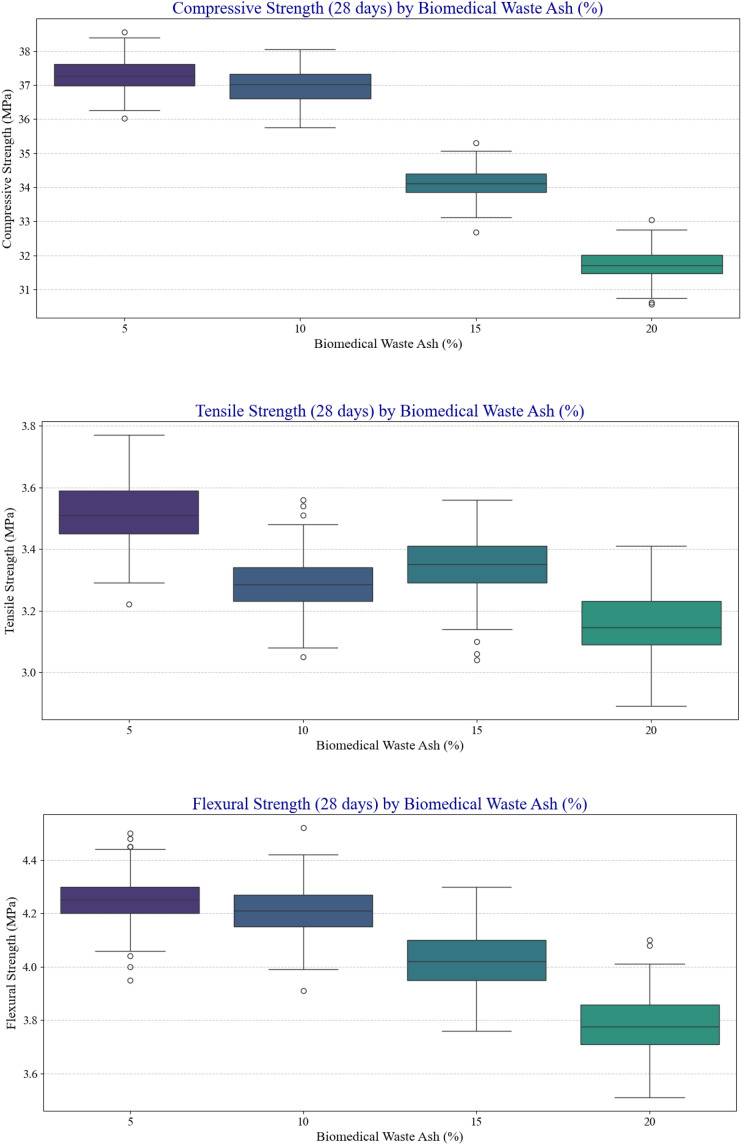
Box plots of CS, TS, FS vs biomedical waste ash (%).

Fig 7 shows the effects of BMWA additions on the compressive, tensile, and flexural strengths of the concrete over 28 days. A consistent downward trend was observed across all three strength parameters, with median values generally decreasing as the percentage of BMWA increases. This indicates a negative correlation between BMWA content and mechanical performance. Although compressive strength has a much more unvarying downward trend, tensile and flexural strengths show smaller variations at intermediate levels of replacement, indicating that a small amount of substitution cannot have a drastic effect on the strength. Despite the presence of outliers reflecting variability, the overall patterns remain evident. Increased levels of replacement of BMWA lead to inferior strength properties, which validate that over-incorporation will have a negative influence on compressive, tensile, and flexural properties. The results suggest that BMWA could prove to be an appropriate partial substitute during the manufacturing of the concrete, but the use of this material should be optimally calculated to ensure a balance between sustainability and the structural needs of concrete.

### 3.4. Radar plot

**Fig 8:**
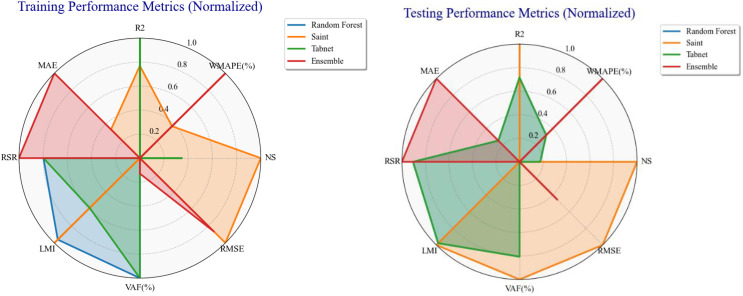
Radar chart for train and test performance metrics.

The radar plot in Fig 8 compares four ML models: RF, Saint, Tabnet, and an Ensemble model based on normalized measures of training performance. The figure of each axis demonstrates the pros and cons of every model, as it is the depiction of a different statistic of assessment, i.e., R^2^, RMSE, MAE, etc. This indicates that the Ensemble model ranks higher in terms of MAE and RSR; RF and TabNet are better in LMI and VAF, whereas SAINT was best in NS and RMSE. The radar plot demonstrates the normalized testing performance measures of the RF, SAINT, TabNet, and Ensemble models. The axes point to different measures, which include R^2^, RMSE, and MAE, in an attempt to depict the comparative performance of the models. SAINT values NS and RMSE the most, whereas TabNet has a consistently good performance at R^2^ and LMI. The ensemble model performed better in MAE and RSR.

### 3.5. 3D surface plot

**Fig 9:**
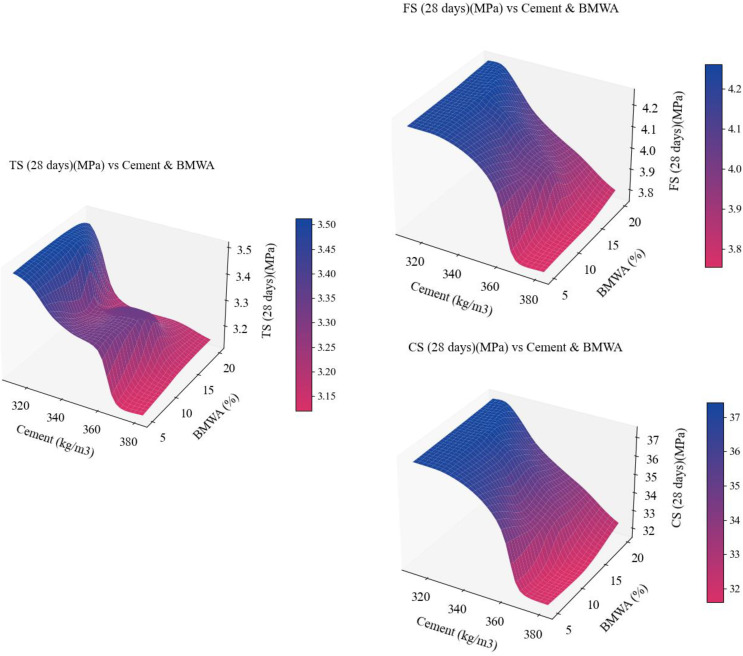
3D surface plots for CS, TS, and FS, respectively.

The 3D surface plots in Fig 9 show the combined effect of cement content (kg/m³) and BMWA% on the mechanical performance of concrete at 28 days, considering CS, TS, and FS. The vertical axis represents the strength values, and the colour scale presents an easy-to-read gradient. The surface representation of the data illustrates that, in general, the strength increases with an increase in the amount of cement added to the mixture; however, an increase in BMWA content beyond a certain level will result in a significant decrease in the performance of the mixture. Therefore, there is a direct relationship between the amount of cement reduced from the original mixture and the amount of BMWA incorporated into the mixture.

A peak in the compressive strength (37 MPa) was achieved by adding more cement to the mixture and reducing the BMWA content; conversely, the strength of the mixture decreases significantly once the BMWA content exceeds 10%. A similar relationship existed in the tensile strength and flexural strength (middle and right) plots, in which the best results were achieved by using mixtures with high amounts of cement and low BMWA content. Although the rate of decline in tensile and flexural strengths appears to be less than the rate of decline in compressive strength, these plots illustrate that to sustain structural performance in mixtures containing BMWA, the ratio of BMWA to total volume of the mixture must be strictly controlled.

### 3.6. Error analysis

**Fig 10:**
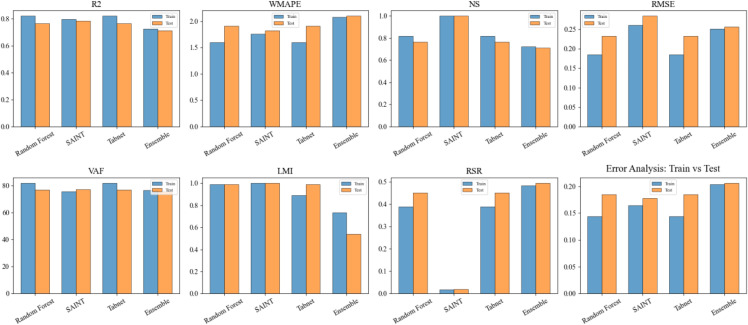
Error Analysis for all the models.

Fig 10 represents an error analysis of the four predicted ML models with parameters: compressive, tensile, and flexural strength of concrete. The models have consistently low error magnitudes relative to the observed strength range, which show that the predictions were very similar to the true results. However, it is possible to see that there was a large difference between the performance of the models. The RF and SAINT models showed the best performance with the lowest error values for both MAE and MSE. The TabNet model produced larger errors for the prediction of compressive strength and, therefore, possibly has some limitations in terms of robustness and could also be overfitting the training data. Overfitting was a common problem with the TabNet model, which may be related to its ability to learn complex interactions in the data.

The low error values seen in the MAE and MSE values of the error distributions, the error distributions indicate stable predictive behavior across the evaluated strength parameters. The majority of the prediction errors were tightly grouped about zero, consistent with the observed error trends across models. The small variability in the prediction errors was confirmed by the compact bars in the plots of the error distributions, which indicate a high level of consistency and a low variance in the predictions. Therefore, the models demonstrate reliable predictive performance within the studied data domain the different strengths of the various concretes. The fact that the models produce consistently low error values for each of the three strengths indicates that the models are versatile and reliable. The analysis confirmed that all four models are suitable for predicting this type of strength, but that the RF and SAINT models would be good options for use in the future due to their lower MAE and MSE values.

### 3.7. Sensitivity analysis

The blue line illustrates that an increase in cement will continue to improve the compressive strength of concrete up to a certain point. The orange illustrates how an increase in BMWA will cause the compressive strength to remain relatively stable only at low replacement levels, not “until zero”. Beyond this point, the compressive strength begins to rapidly decrease. Therefore, moderate BMWA replacement appears to have no detrimental effect on the mechanical properties of the mixture; however, any BMWA replacement beyond the moderate range can have a substantial negative impact on the compressive strength of the mixture due to dilution of cementitious materials.

**Fig 11:**
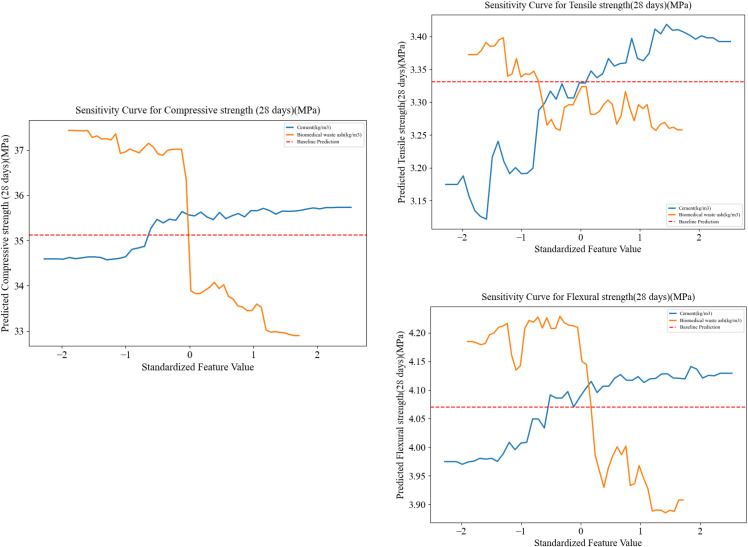
Sensitivity curve for CS, TS, and FS.

The effects of changes in the amounts of cement and BMWA on the tensile and flexural strengths of the mixture. Similar to the effects seen in Fig 11 regarding the compressive strength of the mixture, the tensile and flexural strengths also see improvements as the amount of cement is increased. The use of BMWA had a non-linear effect on the tensile and flexural strengths of the mixture. Therefore, moderate levels of BMWA replacement appear to produce acceptable results in terms of mechanical properties. BMWA replacement rates greater than moderate was likely to negatively affect the tensile and flexural strengths of the mixture. A comparison between the solid black line representing the best-fit model and the red dashed line, which represents the baseline forecast, indicated that BMWA will ultimately negatively affect the tensile and flexural strengths of the mixture.

### 3.8. SHAP analysis

**Fig 12:**
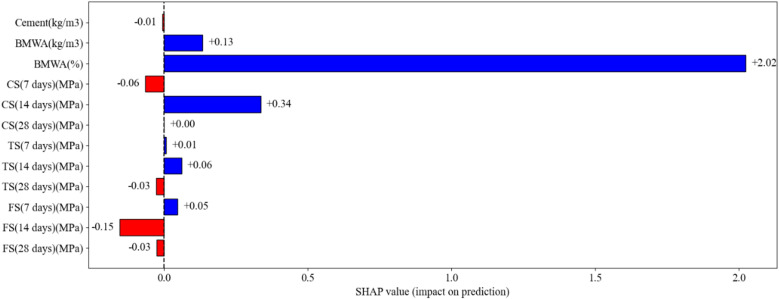
Shap analysis.

The SHAP analysis in Fig 12 displays the feature contribution direction of the trained prediction model for the BMWA concrete; the SHAP analysis used in this section was used for the ML model predicting compressive strength as the output variable and not for the sustainability index or any alternative output variables. The x-axis is a representation of the SHAP values, which indicate both the magnitude and the direction of influence of each input feature on the models’ predictions. Positive SHAP values (blue bars) are indicative of a feature increasing the prediction, while negative SHAP values (red bars) are indicative of a feature reducing it. A key point about SHAP values is that they represent deviation from a baseline prediction and do not reflect a globally monotonic relationship over the entire range of the data. In the chart provided, BMWA (%) is clearly the most important feature contributing to the models’ predictions, with the largest absolute SHAP value indicating a dominant contribution relative to all other features. However, although there exist some localized positive SHAP values (up to +2.02) at low BMWA replacement levels relative to the baseline, these do not indicate an overall strength-enhancing effect. The contribution of BMWA (%) to compressive strength predictions was largely negative at higher replacement levels, as would be expected from true results and correlation analyses. These behaviours reflect the balance between weak filler effects at low BMWA content and strength reductions due to cement dilution at high BMWA content.

The features such as CS (14 days) (MPa) and BMWA (kg/m^3^) had moderate positive and/or negative SHAP contributions, respectively, indicating their role in modifying predictions rather than acting as independent strength-enhancing factors. FS (14 days) (MPa) and FS (28 days) (MPa) contribute negatively, indicating that higher values of flexural strength are associated with lower predicted compressive strength relative to the baseline within the multivariable model context. Interestingly, cement content had a minimal effect, highlighting that the BMWA replacement ratio and not the amount of cement present governs the models’ response. Furthermore, both the water content and the water-binder ratio exhibit negative SHAP contributions throughout, indicating that increased water demand reduces compressive strength, which is consistent with established behaviour of concrete and validates the physically realistic nature of the model. SHAP analysis demonstrated that while BMWA was driving the models’ predictions, strength properties at various curing times modulate the outputs of the model, illustrating the interaction between the chemical and mechanical properties of materials.

#### 3.8.1. SHAP value analysis.

**Fig 13:**
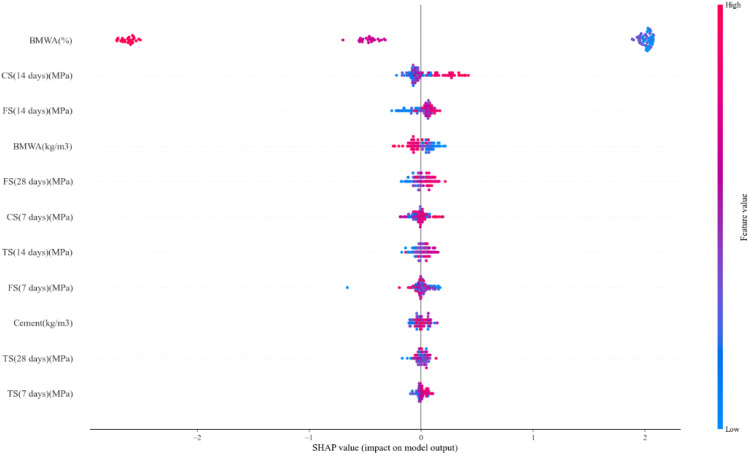
Shap value plot.

The SHAP value trends in Fig 13 show the inter-relationships of the individual features in the data set. As would be expected, there is an extremely high positive relationship (0.96) between the two measures of biomedical waste ash % vs biomedical waste ash kg/m^3^. There is a very high negative relationship (−0.93) between biomedical waste ash % and cement content. This indicates that as the percent replacement by BMWA increases, the cement content decreases. There is also a very high correlation (0.78–0.85) present among the three compressive strength measures at each curing age, which indicates that there was a consistent pattern of development of compressive strength in these concrete mixtures.

There was also evidence of the interdependence of the mechanical properties. The tensile and flexural strengths showed moderate positive correlations (0.57–0.73) to compressive strength, but early age tensile strength (at 7 days) had a low correlation to other strength measures. This suggests that tensile strength may be more variable than other strength measures during the early curing periods. It is evident from the data in this matrix that a negative correlation existed between BMWA % and every strength measure, which further supported the idea that using too much BMWA will result in reduced strength compared to optimal use levels for BMWA. The observation helped to validate the corrected SHAP interpretation and validates the alignment of model explainability and statistical correlation. The matrix presents a visual representation of the “cement-replacement-for-mechanical-performance” trade-off.

### 3.9. Taylor analysis

**Fig 14:**
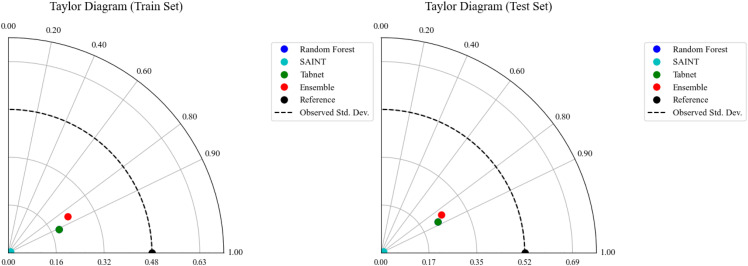
Taylor analysis for train and test values for all models.

The impact of varying percentages of BMWA on the mechanical characteristics of concrete is illustrated in Fig 14. As the percentage of BMWA increases, compressive, tensile, and flexural strength generally decrease because increasing amounts of BMWA dilute the amount of cementitious materials in the mix. Although there appears to be a balance between performance (strength) and sustainability for mixes that contain 10–15% BMWA. The rate at which all strength parameters decrease significantly accelerates at BMWA replacement values greater than 15%, resulting in unacceptable reductions in structural integrity. Therefore, it appears that moderate amounts of BMWA result in acceptable strength retention while also allowing for significant use of sustainable resources.

### 3.10. Pareto front plot

**Fig 15:**
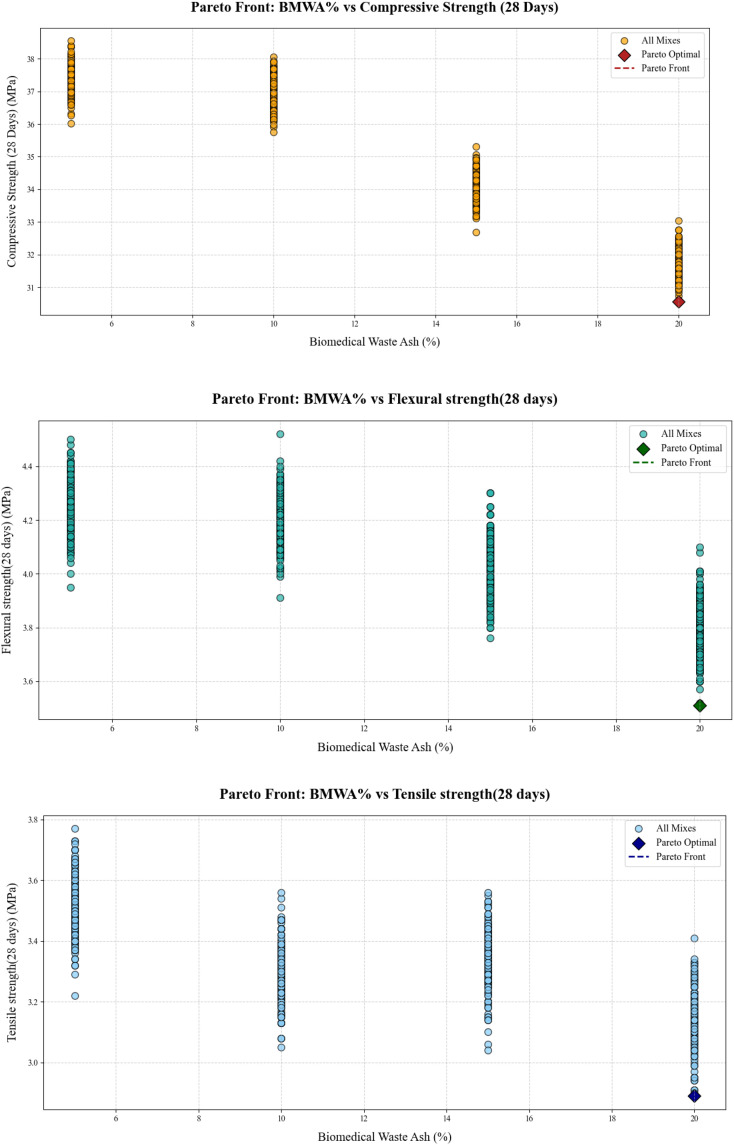
Pareto front plot for CS, FS, and TS, respectively.

The Pareto Front in Fig 15 plots provides a complete visualization of the trade-offs between BMWA% and major mechanical properties of concrete: CS, FS, and TS after 28 days. The performance of different compositions is represented by each plot by a cloud of all possible mix designs in orange. The Pareto Front is drawn in a red dashed line and consists of non-dominated solutions only that provide the optimal balance between the maximization of strength properties and the intensification of the sustainable use of biomedical waste ash. The dark red dots are the best combinations in which any additional addition to the BMWA% would cause a massive decline in the mechanical performance. Such representations enable researchers to ascertain the right proportion of mixes in order to design sustainable concrete without compromising the structural strength.

As the analysis indicates, the mechanical strength qualities reduce when the BMWA% increases, a feature of the nature of the inherent trade-off of adopting various materials. Although there is a relatively modest reduction in CS, this implies that other mixes may be able to maintain reasonable strength values at higher percentages of BMWA. The plots for both FS and TS demonstrate an intermittent behavior and greater sensitivity to BMWA% values. The findings thus provide evidence for the need for multi-objective optimization in designing concrete mixes, i.e., finding a balance between sustainability and structural performance. A Pareto Front provides useful guidance for the selection of mix designs that can be considered as ecologically acceptable yet structurally sound.

### 3.11. Pairwise plot

**Fig 16:**
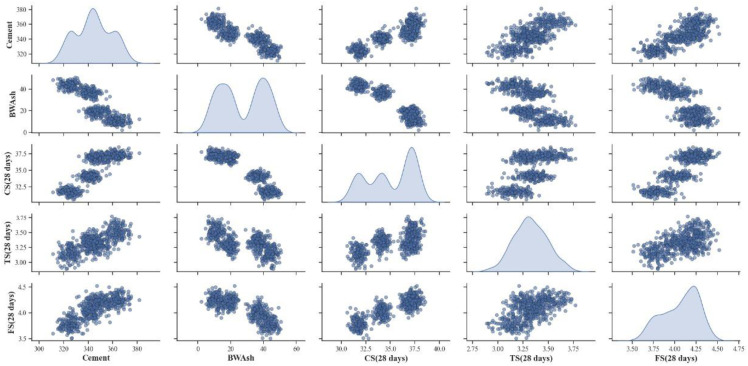
Pairwise plot for CS, FS, and TS, respectively.

The Pairwise Plot shown in Fig 16 represents the five variables, such as cement, BMWA, Compressive strength (CS at 28 days), tensile strength (TS at 28 days), and flexural strength (FS at 28 days). In addition, it is possible to see the pair-wise correlations between the variables through the off-diagonal points, which were displayed by scatter plots. On the other hand, the point along the diagonal shows how each variable is distributed. Both cement and CS at 28 days appear to be distributed along the diagonal; this could mean the data set could have different types of clusters. TS and FS show unimodal distributions, while BMWA showed a left skew. The scatter plots indicate strong positive correlations between cement and the strength parameters (CS, TS, FS), as expected given that increasing cement content normally improves concrete strength. BMWA showed negative correlations with all strength parameters, implying that higher amounts of BMWA reduce strength values. The scatter plots also revealed clustering, suggesting the data may represent different mix designs. The pair plot highlighted the opposing effects of cement (strength enhancer) and BMWA (strength reducer) on the mechanical properties of concrete. These paired plots are intended for exploratory visualization only and are not used to infer standalone causal relationships, which are instead examined using model-based feature importance and SHAP analyses.

### 3.12. External validation

External validation represents a very important part of evaluating predictive models, where the performance of a predictive model that has been developed based upon a set of unseen and independent data sets is evaluated. The goal of this type of evaluation is to determine how well a predictive model will generalize across a wide range of different mixes that can help to identify areas where the model may be overly fitting or insensitive (Refer to Table 4). Unlike internal validation methods that divide a single data set into multiple parts, external validation uses a separate data set that reflects a variety of different mixes. The use of external validation provided a much more realistic measure of predictive framework than internal validation methods in order to make informed decisions about the application of predictive models in a variety of practical engineering applications.

**Table 4:** External validation results.

Cement (kg/m^3^)	Biomedical waste ash (kg/m^3^)	Fine aggregate (kg/m^3^)	Coarse aggregate (kg/m^3^)	Predicted CS (28 days) (MPa)	Predicted TS (28 days) (MPa)	Predicted FS (28 days) (MPa)
312.3620	2.05844	636	1147	31.84099	3.12769	3.769400
485.2142	96.99098	636	1147	31.80321	3.130996	3.766886
419.5981	83.24426	636	1147	31.80994	3.130326	3.767484
379.5975	21.23391	636	1147	31.82564	3.12896	3.768536
246.8055	18.18249	636	1147	31.85747	3.126358	3.77013
246.7983	18.34045	636	1147	31.8574	3.126361	3.770135
217.4250	30.4242	636	1147	31.86605	3.125659	3.770483
459.8528	52.47564	636	1147	31.81093	3.13024	3.767544
380.3345	43.19450	636	1147	31.8216	3.129292	3.76829
412.4217	29.12291	636	1147	31.81981	3.129446	3.768177

The external validation dataset was obtained from an independent peer-reviewed source that was not used during model training, ensuring complete separation from the training dataset. This dataset differed from the training data in terms of mix proportions, BMWA replacement levels, and material combinations, thereby enabling a rigorous assessment of model generalization. The external validation data set contained a variety of new combinations of inputs, including Cement (kg/m³), Biomedical Waste Ash (kg/m³), Fine Aggregate (kg/m³), and Coarse Aggregate (kg/m³), none of which had been included in developing the predictive model. To maintain consistency in methodology and to prevent any form of information leak, each of the preprocessing steps, specifically the feature normalization, was performed solely on the training data set, and then the same preprocessing steps were applied without modification to the external data set. Predictions made by the models were first made in normalized space, and then they were transformed back to the original physical units (MPa) before the predicted values of the output property could be analyzed and reported.

The predicted values of the output properties (i.e., compressive, tensile, and flexural strength at 28 days) exhibited variation around a mean compressive strength value of approximately 31.84 MPa and varied according to the proportion of cement and BMWA after being inversely normalized. The similarity of the predicted values demonstrated that the models continue to respond to the changes in the inputs and provide predictable outcomes when applied to unseen data. The external validation results confirmed that the proposed ML framework is both robust and practical for making predictions regarding the mechanical performance of BMWA-incorporated concrete and for assisting in the data-driven optimization of concrete mix designs in practical engineering applications. Although SAINT exhibited lower pointwise correlation for compressive and tensile strength, its predictions showed reduced variance across unseen data, indicating stable generalization behaviour.

It is evidenced by the limited amount of variance exhibited in the external validation prediction results, since the sample size is so small, the data lie near the center of the normalized feature space of the training data and therefore are being interpolated over. These models are operating in the interpolation regime and have not been asked to perform large amounts of extrapolation in terms of representing unsubstituted levels of BMWA. Similarly, when the normalized predictions were converted from their original unitless format back into physical units (in MPa), the concentration in feature space resulted in very similar values of predicted strength. These findings indicate the models stability in their ability to generalize to new materials that lie in the same substitution domain as those used during training but also suggest that there will be a need for additional validation using datasets that represent a broader range of material compositions and substitution levels.

These similarities among the models provided evidence of how to interpret at the feature level; however, the differences in the two models’ ability to generalize were a function of the architectures of the two models and the characteristics of the dataset. Specifically, the superior ability of the SAINT model to generalize to new samples was due to its use of a transformer-based attention mechanism to identify relevant input features and to minimize the impact of correlations between input variables (noise). There was a distinct advantage to using such a model architecture when dealing with the moderately-sized BMWA substitution dataset. In contrast, the use of ensemble models did not provide a significant benefit to the models ability to generalize since they contained redundant models, redundant decision-making processes, and decision boundaries that overlapped, all of which can lead to an increased bias toward specific datasets. These results suggested that the ability of a model to perform was more dependent upon the suitability of the architecture of the model relative to the characteristics of the dataset than the size of the model.

### 3.13. Sustainability index

The SI analysis’s findings showed that there was a definite trade-off between the BMWA concrete mixes’ mechanical performance, durability improvement, and cement savings. The SI stayed quite tiny (0.023) at lower replacement levels (5%), as the slight decrease in compressive strength almost offset the moderate cement savings and limited durability improvement. The SI improved to 0.057 when the BMWA content was increased to 10%, suggesting that the advantages of durability started to balance the accompanying loss of strength. The SI attained its maximum value of 0.083 with 15% BMWA replacement, which produced the most advantageous result. At this level, the concrete provided the most sustainable mix design by achieving the maximum resistance to chloride ingress while keeping a suitable strength.

Nevertheless, even with the greatest cement savings, raising the BMWA content to 20% further decreased the SI to 0.040 since the significant strength loss outweighed the durability improvements. According to these results, the ideal range for sustainability is between 10 and 15% BMWA, where durability and mechanical performance are best combined to produce structurally sound but environmentally conscious concrete. Durability benefits were assessed from RCPT findings where permeability dramatically decreased up to 15% BMWA, and strength, whose loss values were taken from compressive strength data in your results section, are included in Table 5 of the SI values. Durability gains were derived from reported RCPT trends in the literature and were used here for comparative sustainability assessment rather than direct experimental validation. The specified formula with equal weights is used to normalize SI values.

**Table 5:** SI for BMWA concrete mixes.

BMWA Replacement (%)	Cement Saving (%)	Durability Gain (% vs Control)	Strength Loss (% vs Control)	Sustainability Index (SI)
0 (Control)	0	0	0	0.00
5	5	6	4	0.023
10	10	15	8	0.057
15	15	22	12	0.083
20	20	10	18	0.040

## 4. Implications of the study

BMWA can be considered as a sustainable alternative to cement for use in concrete in order to reduce dependence on the highly energy intensive process of producing clinker, thereby contributing to solving two significant problems, including the disposal of medical waste and the reduction of greenhouse gases through the creation of less environmentally impactful products [62,63]. Thus, BMWA provides the opportunity to create concrete that will incorporate the dual benefits of waste valorization and the production of materials with a lower environmental footprint that aligns with the principles of the circular economy and will support the pursuit of global sustainability. The positive results obtained from testing durability at relatively low replacement ratios demonstrate the potential for the use of BMWA in applications where resistance to chloride intrusion and permeability are critical.

The research demonstrated the utility of advanced machine learning algorithms as predictive tools for physical technologies. Through the use of SHAP and sensitivity analyses, the ability of the model to be interpreted will increase further with the addition of RF and TabNet engines. Engineers will have confidence in making predictions about the performance of the engine and subsequently have the knowledge base to select the optimal percentage of replacement for BMWA. An additional major component of the theoretical underpinnings of this methodology is a scalable method for determining the best combination of ingredients (i.e., mix design) and assessing the quality of those ingredients. This scalable methodology will significantly reduce reliance on time consuming and resource intensive experimental trial-and-error experimentation methods. The technological advancements resulting from this research have implications for a societal perspective and the environment. In addition to providing a viable and economically beneficial means of managing hazardous waste by incorporation into building construction practices, the inclusion of BMWA in the production of building materials will result in savings by decreasing the quantity of cement required and improving durability. Moreover, the implementation of sustainable approaches to engineering practice will contribute to increased resilience in the development of infrastructure and assist in achieving SDG’s 11, 12 and 13.

SI has provided researchers and practitioners with a method for easily incorporating BMWA into concrete. The SI provides a means for practitioners to evaluate the most sustainable replacement levels of BMWA based upon durability, mechanical performance, and environmental considerations. The results clearly indicate that the greatest sustainability occurs when 10–15% of BMWA is replaced. The findings clearly demonstrate that replacing 10–15% of BMWA balances both durability improvement and the goals of the circular economy. BMWA-based concretes can be strategically employed in applications that are maritime, where improved durability is more critical than maximum compressive strength. The SI approach enabled the creation of a reproducible evaluation tool for future research using additional non-traditional binders and thereby set the stage for developing standardized sustainability evaluations for all cementitious materials studies.

## 5. Conclusion

The study found that BMWA can be used to replace cement in concrete. The replacement of cement by BMWA has the advantage of creating a double effect: it reduces the amount of cement needed to produce a given quantity of concrete, and also contributes to the disposal of biomedical wastes. ML was employed to develop models capable of predicting the strength of BMWA concrete; these ML models demonstrated high predictive capability relative to the literature-derived properties of the tested materials. The best performing models among those examined were RF and TabNet, but all models had sufficient ability to generalize from the training set to the test set; therefore, the use of deep learning-based methods is valid for modelling concrete behaviour, even if the data sets are limited. There was no improvement in prediction quality, which was observed using Ensemble Methods compared to the individual models. Therefore, it appears that the use of specialized models for specific material types is critical for achieving accurate predictions from data for such materials.

A significant correlation was found in the study between the substitution level of BMWA and both the strength and durability of the resulting concrete. The increasing levels of BMWA substitution resulted in decreased strength of the concrete (both tensile and compressive). Moderate levels of BMWA substitution (10–15%) have been shown to provide significantly improved durability. These results indicate that the incorporation of appropriate amounts of BMWA into the mixture can provide the necessary balance between retained strength and improved durability, thereby providing the opportunity to create sustainable design options for concrete. Sensitivity and SHAP analyses were performed to determine which factors affect the strength predictions made by the models, and it was determined that BMWA is the primary factor affecting strength prediction, and therefore must be precisely controlled. The SI analysis indicated that the optimal range of BMWA substitution is between 10–15% (with a peak at 15% substitution), and thus provides a basis for optimizing sustainable mixture designs.

The literature data suggest that, at optimized replacement levels, BMWA incorporation is associated with reduced permeability and improved durability, which are indicative of potential immobilization of hazardous constituents under typical service conditions. The observed reductions in permeability at optimized BMWA replacement levels suggest a potential reduction in transport pathways for hazardous constituents; however, definitive assessment of environmental safety requires dedicated leaching and long-term exposure studies. BMWA-modified concrete may be considered for non-prestressed and durability-critical applications where resistance to chloride ingress is prioritized over maximum compressive strength, subject to further environmental and field-scale validation.

Future studies would be beneficial to conduct large-scale experimental programs to increase the size of the datasets and capture greater variability in the different mix designs and curing conditions used. The limitation of the study is the lack of information regarding the microstructure and chemistry of the BMWA incorporated into the concrete, which limits the ability to directly assess the reactivity of the BMWA and the phases formed during hydration.

The developed ML models are intended for predictive and decision-support purposes and do not replace mechanistic or microstructural characterization of BMWA interactions. Future studies should compare advanced ML techniques with conventional baselines to better understand their capabilities and limitations, and incorporate life-cycle assessments and cost-benefit analyses to enable informed decision-making. The pilot projects and field trials will provide additional evidence for the feasibility of scaling up the use of BMWA in sustainable construction and aligning with the principles of the circular economy and global sustainability goals. The study provides one of the first integrated frameworks combining advanced ML techniques, interpretability analysis, and sustainability indexing for BMWA concrete to provide a data-driven and scalable path to circular economy-based construction practices.

## Supporting information

S1 File Biomedical waste ash dataset 600. (XLSX)
